# *miR-519a* enhances chemosensitivity and promotes autophagy in glioblastoma by targeting STAT3/Bcl2 signaling pathway

**DOI:** 10.1186/s13045-018-0618-0

**Published:** 2018-05-29

**Authors:** Hong Li, Lei Chen, Jun-jie Li, Qiang Zhou, Annie Huang, Wei-wen Liu, Ke Wang, Liang Gao, Song-tao Qi, Yun-tao Lu

**Affiliations:** 1 Department of Neurosurgery, Shanghai Tenth People’s Hospital, Tongji University School of Medicine, Shanghai, 200072 People’s Republic of China; 2 Department of Neurosurgery, Nanfang Hospital, Southern Medical University, Guangzhou, 510515 Guangdong Province People’s Republic of China; 3 Nanfang Neurology Research Institution, Nanfang Hospital, Guangzhou, 510515 Guangdong Province People’s Republic of China; 4 Nanfang Glioma Center, Guangzhou, 510515 Guangdong Province People’s Republic of China; 5 Brain Tumor Research Center, The Hospital for Sick Children, Toronto, Canada; 6 Department of Plastic and Aesthetic Surgery, Nanfang Hospital, Southern Medical University, Guangzhou, 510515 Guangdong Province People’s Republic of China

**Keywords:** *miR-519a*, Signal transducer and activator of transcription 3, Glioblastoma, Autophagy, Chemoresistance

## Abstract

**Background:**

Chemoresistance to temozolomide (TMZ) is a major challenge in the treatment of glioblastoma (GBM). We previously found that *miR-519a* functions as a tumor suppressor in glioma by targeting the signal transducer and activator of transcription 3 (STAT3)-mediated autophagy oncogenic pathway. Here, we investigated the effects of *miR-519a* on TMZ chemosensitivity and autophagy in GBM cells. Furthermore, the underlying molecular mechanisms and signaling pathways were explored.

**Methods:**

In the present study, two stable TMZ-resistant GBM cell lines were successfully generated by exposure of parental cells to a gradually increasing TMZ concentration. After transfecting U87-MG/TMZ and U87-MG cells with *miR-519a* mimic or inhibitor, a series of biochemical assays such as MTT, apoptosis, and colony formation were performed to determine the chemosensitive response to TMZ. The autophagy levels in GBM cells were detected by transmission electron microscopy, LC3B protein immunofluorescence, and Western blotting analysis. Stable knockdown and overexpression of *miR-519a* in GBM cells were established using lentivirus. A xenograft nude mouse model and in situ brain model were used to examine the in vivo effects of *miR-519a*. Tumor tissue samples were collected from 48 patients with GBM and were used to assess the relationship between *miR-519a* and STAT3 expression.

**Results:**

TMZ treatment significantly upregulated *miR-519a* in U87-MG cells but not in U87-MG/TMZ cells. Moreover, the expression of *miR-519a* and baseline autophagy levels was lower in U87-MG/TMZ cells as compared to U87-MG cells. *miR-519a* dramatically enhanced TMZ-induced autophagy and apoptotic cell death in U87-MG/TMZ cells, while inhibition of *miR-519a* promoted TMZ resistance and reduced TMZ-induced autophagy in U87-MG cells. Furthermore, *miR-519a* induced autophagy through modification of STAT3 expression. The in vivo results showed that *miR-519a* can enhance apoptosis and sensitized GBM to TMZ treatment by promoting autophagy and targeting the STAT3/Bcl-2/Beclin-1 pathway. In human GBM tissues, we found an inverse correlation between *miR-519a* and STAT3 expression.

**Conclusions:**

Our results suggested that *miR-519a* increased the sensitivity of GBM cells to TMZ therapy. The positive effects of *miR-519a* may be mediated through autophagy. In addition, *miR-519a* overexpression can induce autophagy by inhibiting STAT3/Bcl-2 pathway. Therefore, a combination of *miR-519a* and TMZ may represent an effective therapeutic strategy in GBM.

**Electronic supplementary material:**

The online version of this article (10.1186/s13045-018-0618-0) contains supplementary material, which is available to authorized users.

## Background

Glioblastoma (GBM) is the most common primary malignant brain tumor in adults [1]. Multimodality treatment such as cytoreductive surgery followed by radiotherapy with concomitant and adjuvant temozolomide (TMZ) chemotherapy has been widely accepted as the new standard of care for patients with newly diagnosed GBM. However, the prognosis of TMZ-treated patients remains dismal, with a median survival of 12.1–14.6 months [2–4]. Intrinsic or acquired chemoresistance to TMZ is a major clinical obstacle for the treatment of GBM patients. Therefore, a better understanding of the molecular mechanisms underlying TMZ chemoresistance may lead to improved clinical outcomes in GBM patients.

Recently, several studies have shown that anticancer therapies can induce autophagy, which constitutes a novel mechanism of chemoresistance in cancer [5–8]. Autophagy is a highly evolutionarily conserved process that occurs in virtually all eukaryotic cells and has been implicated in various physiological and pathological conditions [9]. In some cases, autophagy induces apoptotic death in GBM cells upon TMZ treatment, and treatment with autophagy inducer rapamycin can further enhance chemotherapy-induced apoptosis [10–14]. In other cases, TMZ-induced autophagy may delay cell death [15–17]. Therefore, modulation of autophagy in response to TMZ treatment may hold great promise for circumventing chemotherapeutic resistance and improving anticancer efficacy in GBM patients. However, the roles of autophagy in regulating GBM cell death and survival remain controversial.

MicroRNAs (miRs) are small, non-coding RNA molecules (20–22 nucleotides in length) that negatively regulate gene expression by binding to the 3′-untranslated region (3′UTR) of target mRNAs [18]. miRs have been shown to be key players in a wide range of biological processes, including proliferation, apoptosis, and migration [19]. Recent evidence has indicated that miRs can regulate the chemosensitivity of glioma cells to TMZ by modulating autophagy signaling [20]. Previously, we demonstrated that *miR-519a* is closely related to improved prognosis of GBM patients [21]. However, the molecular mechanisms underlying the role of *miR-519a* in the chemoresistance of GBM remain unclear.

Signal transducer and activator of transcription 3 (STAT3) functions as a signal messenger and transcription factor, which regulates the transcription of downstream target genes during malignant transformation and tumor development. Several studies have demonstrated that STAT3 overexpression in glioma cells can promote tumor progression [22–24]. A growing body of evidence has implicated STAT3 in the regulation of autophagy, from the assembly of autophagosomes to their maturation [25]. In addition, differential localization of STAT3 may regulate autophagy in distinct ways [25]. For instance, nuclear STAT3 may upregulate BCL2 expression and lead to autophagy inhibition [26]. Therefore, a better understanding of the role of STAT3 signaling in regulating autophagy may provide new insights into the mechanisms of chemoresistance and the potential strategies to overcome TMZ chemoresistance in GBM.

In the present study, we evaluated whether *miR-519a* can affect the chemosensitivity of TMZ in GBM. Furthermore, the roles of *miR-519a* in the modulation of autophagy via STAT3/Bcl-2/Beclin-1 signaling pathway were investigated.

## Methods

### Cell lines and reagents

U87-MG cells were obtained from the Cell Bank of the Chinese Academy of Sciences (Shanghai, China) and were cultured in Dulbecco’s modified Eagle’s medium (DMEM) with 10% fetal bovine serum (FBS; Gibco, Carlsbad, CA, USA), 100 U/mL penicillin, and 100 mg/mL streptomycin (Gibco) at 37 °C in a humidified incubator with 5% CO_2_. The methods for culturing patient-derived GBM cell line G131212 were described previously [21]. TMZ-resistant cell lines were generated by iterative pulse exposure of U87-MG and G131212 GBM cells to TMZ. The derived resistant cell lines were designated as U87-MG/TMZ and G131212/TMZ, respectively. Meanwhile, a stock solution of TMZ (100 mM; cat. no. T2577; Sigma-Aldrich, St. Louis, MO, USA) was dissolved in dimethylsulfoxide (DMSO; cat. no. D2650; Sigma-Aldrich) and stored at − 20 °C. 3-Methyladenine (3-MA; cat. no. M9281; Sigma-Aldrich) was prepared freshly in DMEM at 60 °C and then diluted to 5 mM before use.

### Oligonucleotides and siRNA transfection

The miRNA mimic, miRNA inhibitor, STAT3 siRNA, and scrambled siRNA were synthesized by RiBoBio (China). The oligonucleotide sequences were listed in Table S1 (Additional file 1: Table S1). The miRNA overexpression vector pCMV-MIR519A (MI0003182) and the empty vector control were obtained from OriGene (Rockville, MD, USA). The *miR-519a* sponge and empty vector control were purchased from GeneChem (China). Transfections were performed using Lipofectamine 2000 reagent (cat. no. 11668-019; Invitrogen, Carlsbad, CA, USA) according to the manufacturer’s instructions.

### Cell viability assay

Cell viability was assessed by using MTT assays. First, cells were seeded in 96-well plates at a density of 8000 cells per well. After an overnight incubation, the cells were treated under the indicated conditions. At the end of the treatment, 0.5 mg/mL MTT was added to each well and incubated for 4 h. Then, the supernatants were aspirated carefully, and formazan crystals were dissolved in DMSO. Finally, the absorbance was measured at 550 nm using Thermo Varioskan Flash reader (Thermo Fisher Scientific, Waltham, MA, USA).

### Colony forming cell assay

Cells (200 cells/well) were seeded onto 6-well culture plates and cultured in DMEM supplemented with 10% FBS. The cells were treated with the indicated agents and incubated for 10–14 days at 37 °C and 5% CO_2_. Colonies were then stained with 0.1% crystal violet (Sigma-Aldrich) and counted. In some experiments, cells were pre-treated for 1 h with 3-MA (Sigma-Aldrich) or rapamycin (Abcam, San Francisco, CA, USA), followed by an incubation period of 24 h. For each set of clones, three independent assays were carried out.

### Cell apoptosis assay

GBM cells were transfected with miRNAs (or anti-miRNAs) and/or incubated with 400 μM TMZ for 36 h. Subsequently, cells were harvested and stained with propidium iodide (PI) and annexin V-fluorescein isothiocyanate (FITC) for apoptotic analysis. The percentage of apoptotic cells was calculated as the sum of early and late apoptotic cells located in the lower and upper right quadrants, respectively.

### Green fluorescent protein-LC3 puncta assay

GBM green fluorescent protein (GFP)-LC3 stable cells were transfected with miRNAs or anti-miRNAs. Two days after the transfection, cells were fixed with 4% paraformaldehyde. GFP-LC3 dot formation was observed under a confocal laser scanning microscope (FLUOVIEW FV10i; Olympus, Japan). The average number of GFP-LC3 dots/cell was counted in at least 200 cells.

### Transmission electron microscopy (TEM)

GBM cells were subjected to different treatments. The freshly harvested tumors from mice were fixed overnight with 2.5% glutaraldehyde at 4 °C and post-fixed in 1% osmic acid. The fixed samples were then dehydrated using a graded series of ethanol (70–100%) and embedded in EPON resin. Ultrathin sections were cut with an ultramicrotome and double-stained with uranyl acetate and lead citrate. The stained sections were then examined using a TEM (H-7650; Hitachi, Tokyo, Japan).

### Construction of stable lentiviral clones

Lentiviral expressing GFP empty vector (NC-LV), GFP vector overexpressing *miR-519a* (LV-*miR-519a*), or GFP vector inhibiting *miR-519a* expression (LV-anti-*miR-519a*) was constructed by Systems Biosciences Inc. (Mountain View, CA, USA). Virus production and cell transduction in GBM cells were performed as previously described [21], and cells were selected in puromycin (1 μg/mL). The selected cells were sorted by flow cytometry to maintain a GFP-positive rate for at least 95%.

### Western blotting

Western blotting was performed as described previously [27]. The antibodies including anti-LC3B (cat. no. 4445), anti-BECN1/Beclin (cat. no. 3495), anti-STAT3 (cat. no. 12640), anti-phospho-STAT3 (Tyr705; cat. no. 9145), and anti-CASP3/caspase-3 (cat. no. 9915) were purchased from Cell Signaling Technology, while anti-Bax (cat. no. sc-7480) and anti-Bcl-2 (cat. no. sc-509) were obtained from Santa Cruz Biotechnology, Santa Cruz, CA, USA.

### RNA isolation and quantitative reverse transcription polymerase chain reaction

Total miRNA from cultured cells was extracted using TRIzol reagent (cat. no. 15596-026; Invitrogen), and the RNA purity was evaluated by A260/A280 ratio of 1.9–2.0. cDNA was synthesized from 1 μg of total RNA using PrimeScript RT reagent kit (cat. no. RR047A; Takara, Shiga, Japan). The primers used for PCR amplification were listed in Table S2 (Additional file 2: Table S2). The expression levels of target genes were quantified on a Stratagene Mx-3005p instrument (Agilent Technologies Inc., USA) by using Maxima SYBR Green/ROX qPCR Master Mix (cat. no. K0222; Thermo Scientific). Triplicate samples were examined.

### Tumor xenograft assays in nude mice

BALB/c nude mice (4–5 weeks old) were provided by the Experimental Animal Center of Southern Medical University. U87-MG cells with stable expression of lentivirus *miR-519a* or U87-MG/TMZ cells with stable expression of *miR-519a* shRNA were injected into the left flank of the mice, while control cells were injected into the right flank of the mice. Mice were injected intraperitoneally (i.p.) with phosphate-buffered saline alone (control) or TMZ (Merck Co., NJ, USA; 20 mg/kg/mouse) once every other day for 3 weeks, starting on day 3. Tumor volume and animal weight were assessed every 4 days. Tumor volume was calculated using the following formula: volume (mm^3^) = 4/3 × 3.14 × radius (mm)^3^. Mice were humanely sacrificed on day 24. Tissue blocks were subjected to immunohistochemical staining and TEM analysis.

For survival analysis in the orthotopic xenograft model, nude mice were randomly divided into four groups: LV-anti-*miR-519a* group, LV-anti-NC group, LV-anti-NC+TMZ group, and LV-anti-*miR-519a*+TMZ group. In this model, 3 × 10^5^ cells were stereotactically implanted into the right striatum of the mice. Twenty four days after injection, tumor burden of mice was assessed using magnetic resonance imaging (MRI) scanner (Bruker Medical Inc., Billerica, MA, USA). The number of surviving nude mice was recorded, and survival analysis was performed by Kaplan-Meier survival curves.

### Patient samples

In this study, 24 patients with recurrent GBM treated with TMZ before second surgery and 24 patients with primary GBM without TMZ treatment were recruited from Nanfang Hospital (Guangzhou, China). Tissue samples were retrieved from the Department of Pathology and subjected to sectioning process. Subsequently, the tissue sections were fixed and immunohistochemically stained with anti-LC3B anti-STAT3, and anti-CASP3/caspase-3 antibodies (cat. no. 4445, 12649, and 9915, respectively; Cell Signaling Technology, Danvers, MA, USA).

### Statistical analysis

All experiments were performed in triplicate and repeated at least once. All data were expressed as means ± standard deviation. If the homogeneity of variance assumption was met, one-way analysis of variance (ANOVA) was performed to determine the differences between groups, while least significant difference (LSD) test was used to compare the means of two groups. If the variance was heterogeneous, Welch test was applied to compare the differences between groups, while Dunnett’s T3 test was used for pairwise comparisons. *p* values of less than 0.05 were considered statistically significant.

## Results

### miR-519a sensitized GBM cells to TMZ treatment

**Fig. 1:**
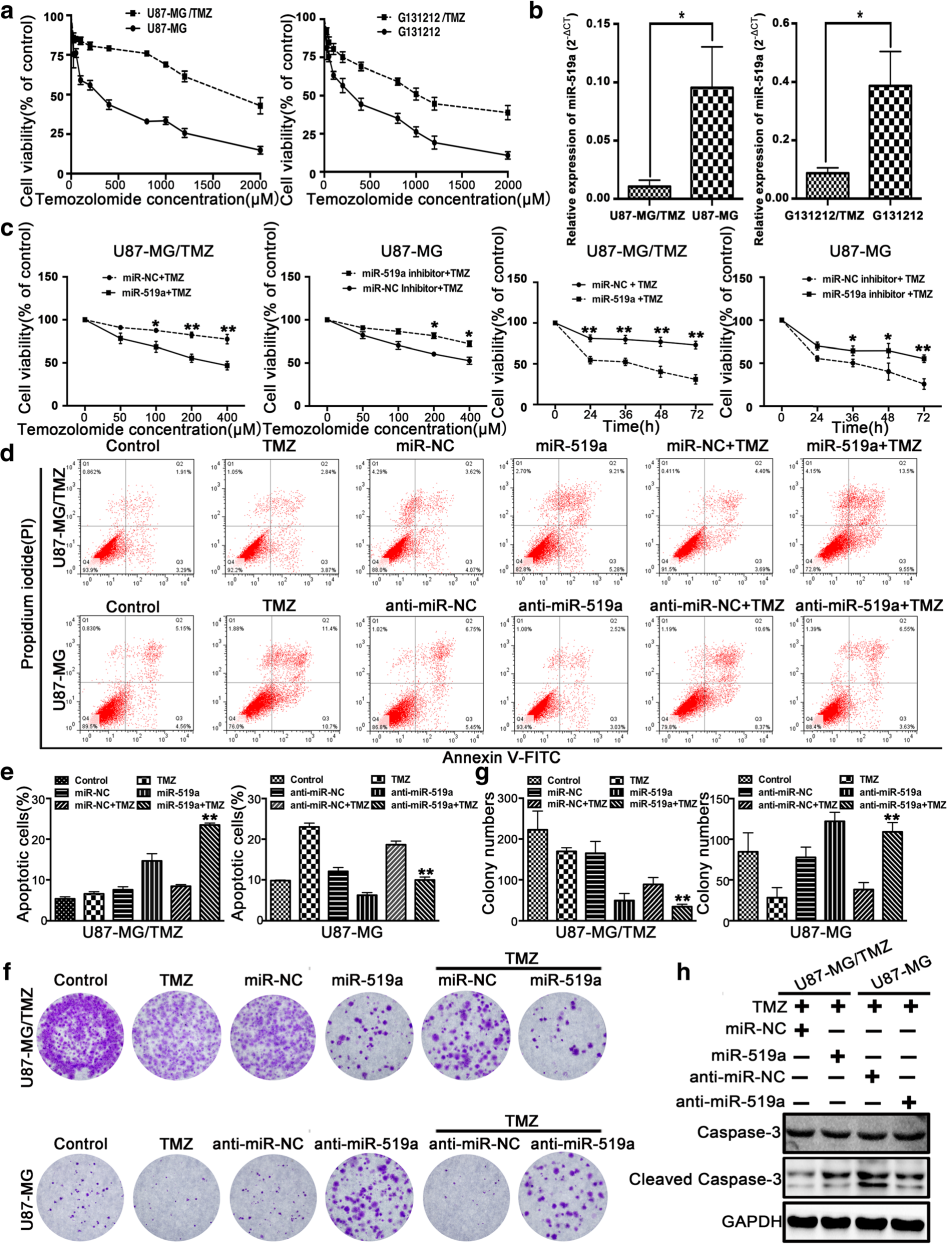
*miR-519a* sensitized GBM cells to TMZ treatment. **a** Parental (black ellipses) or resistant (black squares) cells were exposed to increase the concentrations of TMZ in serum-free medium (acute growth inhibition assay) for 72 h. Cell viability was measured by MTT assays. **b** The expression of *miR-519a* was detected by qRT-PCR. **c** Cell viability of DMSO- or TMZ-treated GBM cells transfected with *miR-519a* or anti-*miR-519a*. **d**, **e** GBM cells transfected with *miR-519a* or anti-*miR-519a* were treated with 400 μM TMZ for 36 h. Cells were harvested and stained with PI and annexin V-FITC for apoptotic analysis (**p < 0.01 vs. TMZ group). **f**, **g** Colony formation assay showing the sensitizing effects of *miR-519a* on GBM cells after TMZ treatment (**p < 0.01 vs. TMZ group). **h** Western blot analysis of cleaved caspase-3 in GBM cells transfected with *miR-519a* or anti-*miR-519a* and then treated with TMZ for 24 h. Data represent the mean (± standard deviations) and are representative of three independent experiments, each performed in triplicate. *p < 0.05, **p < 0.01

A stable TMZ-resistant phenotype of parental U87-MG cells (a GBM cell line) and G131212 cells (patient-derived GBM cells) was established by repetitive pulse exposure to increasing concentrations of TMZ for 6 months [28]. Both TMZ-resistant U87-MG/TMZ and G131212/TMZ cells exhibited lower sensitivity to TMZ and lower proliferation doubling times than the respective parental cells (Fig. 1a and Additional file 3: Figure S1).

To determine the effect of *miR-519a* on the chemosensitivity of TMZ, we first examined the expression levels of *miR-519a* in resistant sublines. As shown in Fig. 1b, the expression of *miR-519a* was lower in the resistant U87-MG/TMZ and G131212/TMZ cells compared to their respective parental sensitive cells. Moreover, TMZ may induce the expression of *miR-519a* in U87-MG cells but not in U87-MG/TMZ cells (Additional file 4: Figure S2).

To further confirm the role of *miR-519a* in TMZ chemoresistance of GBM cells, we transiently transfected U87-MG (or U87-MG/TMZ) cells with *miR-519a* inhibitor (or *miR-519a* mimic) and evaluated the effects of miR by using MTT and clonogenic assays. We found that *miR-519a* sensitized the U87-MG/TMZ cells to TMZ and confirmed that anti-*miR-519a* induced TMZ resistance in U87-MG cells (Fig. 1c). The results of flow cytometry analysis revealed that *miR-519a* overexpression significantly increased GBM cell apoptosis caused by TMZ treatment, whereas downregulation of *miR-519a* inhibited TMZ-induced apoptosis in U87-MG cells (Fig. 1d, e). Colony formation in U87-MG/TMZ cells was markedly lower than that in the control group, while higher colony formation was found in U87-MG cells (Fig. 1f). These results were validated by using the overexpressing vector pCMV-MIR-519a or the inhibition vector *miR-519a* sponge (Additional file 5: Figure S3).

Furthermore, Western blot analysis showed that TMZ-induced cellular apoptosis was greatly enhanced by *miR-519a* compared to NC, while knockdown of endogenous *miR-519a* decreased TMZ-induced cell apoptosis (Fig. 1h). Moreover, *miR-519a* can effectively sensitize GBM cells to irradiation treatment (Additional file 6: Figure S4). Collectively, these data supported that *miR-519a* may enhance TMZ chemosensitivity in GBM cells.

### miR-519a promoted TMZ-induced autophagy in GBM cells

**Fig. 2:**
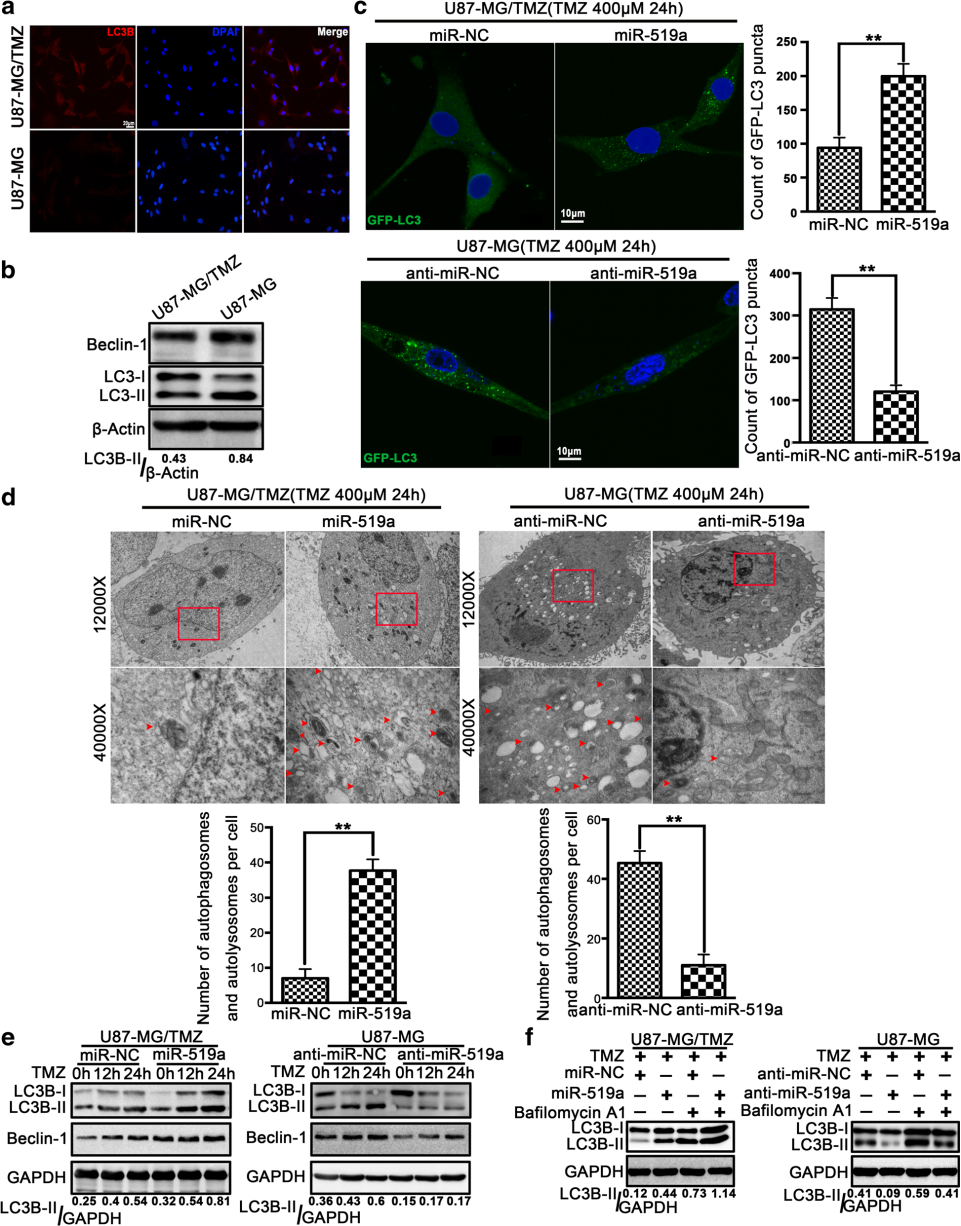
*miR-519a* enhanced TMZ-induced autophagy in GBM cells. The expression levels of LC3-II in U87-MG/TMZ and U87-MG cells were evaluated by immunofluorescence assays (**a**) and Western blotting (**b**). Red indicates LC3B and blue indicates nuclei. **c** Both U87-MG/TMZ and U87-MG cells were transfected with GFP-LC3 construct expressing either *miR-519a* or anti-*miR-519a*, followed by treatment with 400 μM TMZ. The numbers of GFP-LC3 puncta were quantified using confocal laser scanning microscopy. **d** Both U87-MG/TMZ and U87-MG cells were transfected with either *miR-519a* or anti-*miR-519a* for 24 h, followed by treatment with 400 μM TMZ. Cell samples were prepared for transmission electron microscopy analysis. The arrows indicate autophagic vacuoles. **e** U87-MG/TMZ cells transfected with *miR-519a* and parental U87-MG cells transfected with anti-*miR-519a* were exposed to 400 μM TMZ at different time points (0–24 h). Whole cell lysates were analyzed by Western blotting. **f** U87-MG/TMZ cells transfected with or without *miR-519a* and treated with or without 400 μM TMZ after incubation with 20 nM bafilomycin A1 for 2 h. U87-MG cells transfected with or without anti-*miR-519a* were treated with 20 nM bafilomycin A1 for 2 h. Cell lysates were analyzed by Western blotting. *p < 0.05, **p < 0.01

The results of both immunofluorescence (Fig. 2a) and Western blotting (Fig. 2b) showed that U87-MG/TMZ cells had lower autophagic activity than U87-MG cells. Meanwhile, the sensitivity of both TMZ-sensitive and TMZ-resistant cells was tested, with regard to autophagy inducers, including low glucose (LG), chloroquine (CQ), and rapamycin (Rapa) at different concentrations. These results indicated that U87-MG cells were sensitive to all the tested concentrations of rapamycin, whereas the growth of U87MG/TMZ cells was not affected by rapamycin (Additional file 7: Figure S5). It is thereby proposed that the lack of autophagy is a possible mechanism for TMZ resistance.

In order to determine the effects of *miR-519a* on autophagy induction following TMZ treatment, U87-MG/TMZ cells were transfected with *miR-519a* and U87-MG cells were transfected with anti-*miR-519a* prior to TMZ treatment. In addition, GFP-LC3 was stably expressed in U87-MG and U87-MG/TMZ cells to facilitate the visualization of autophagy. As compared with control cells, overexpression of *miR-519a* enhanced TMZ-induced GFP-LC3 puncta formation in U87-MG/TMZ cells, whereas knockdown of *miR-519a* in U87MG cells inhibited the formation of TMZ-induced GFP-LC3 puncta (Fig. 2c). Besides, TEM was used to count the number of autophagic vacuoles per cell. The results revealed that the number of autophagic vacuoles per cell was markedly increased in *miR-519a*-overexpressing U87-MG/TMZ cells and decreased in *miR-519a*-knockdown U87-MG cells after TMZ treatment (Fig. 2d).

In order to detect the occurrence of autophagy after TMZ treatment in the presence or absence of *miR-519a*, we conducted Western blot analysis to examine the levels of two autophagy-related proteins: LC3B and Beclin-1. As shown in Fig. 2e, the expression levels of LC3-II and Beclin-1 proteins were increased in *miR-519a*-overexpressing U87-MG/TMZ cells. On the other hand, knockdown of *miR-519a* inhibited the expression of LC3-II and Beclin-1 in U87-MG cells. Since the increased LC3-II may be due to either autophagy induction or inhibition of autophagic flux [29], BafA1, an inhibitor of fusion between autophagosomes and lysosomes, was used in this study. Twenty-four hours after TMZ treatment, the BafA1-treated negative control cells displayed markedly increased accumulation of LC3II, and the ectopic expression of miR-519a may enhance these effects (Fig. 2f). Taken together, our data indicated that the inductive effects of miR-519a on autophagy can be resulted from the induction of early stages of autophagy, rather than from the suppression of autophagosome degradation.

### miR-519a sensitized GBM cells to TMZ treatment by promoting autophagy

**Fig. 3:**
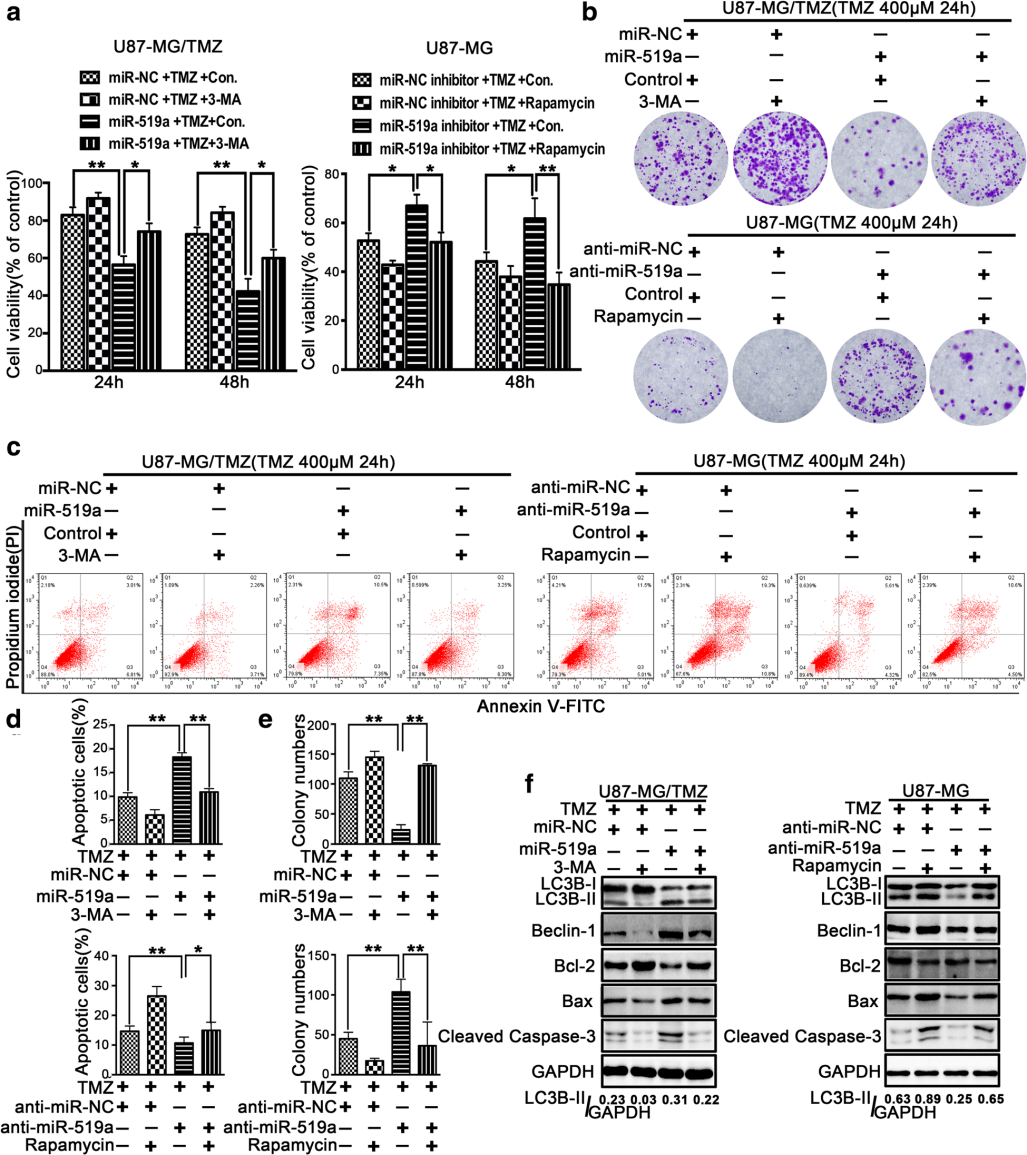
*miR-519a* sensitized GBM cells to TMZ treatment partly regulated by autophagy. U87-MG/TMZ cells transfected with *miR-519a* were treated with or without 400 μM TMZ after incubation with 3-MA (an inhibitor of autophagy) for 2 h. U87-MG cells transfected with anti-*miR-519a* were treated with or without 200 nM rapamycin for 2 h. The cells were then analyzed for the following: **a** assessment of proliferation by MTT assay; **b**, **e** assessment of colony formation; **c**, **d** assessment of apoptosis by FACS analysis of PI-stained cells; and **f** assessment of caspase-3 activity. *p < 0.05, **p < 0.01

Both 3-MA and rapamycin were not able to affect the viability of U87MG and U87MG/TMZ cells, respectively, without TMZ treatment. Indeed, pre-treatment of U87-MG cells with 3-MA (an inhibitor of autophagy) significantly attenuated TMZ-induced cytotoxicity, while rapamycin enhanced TMZ cytotoxicity in U87-MG/TMZ cells (Additional file 8: Figure S6). These results further suggested that autophagy may contribute to the chemosensitivity of TMZ in GBM cells. To confirm whether autophagy is responsible for the cellular sensitization during TMZ chemotherapy enhanced by *miR-519a*, we assessed the cell apoptosis rate after the inhibition and induction of autophagic activity in both U87-MG/TMZ and U87-MG cells, respectively. Treatment with 3-MA significantly attenuated the anti-proliferative effects of *miR-519a* in miR-519a-overexpressing U87-MG/TMZ cells, whereas rapamycin treatment reversed the chemoresistance of TMZ in U87-MG cells transfected with anti-*miR-519a*, as indicated by MTT and colony formation assays, respectively (Fig. 3a, b, e).

The results of flow cytometry assays clearly showed that 3-MA significantly attenuated TMZ-induced apoptosis in *miR-519a*-overexpressing U87-MG/TMZ cells, whereas rapamycin enhanced TMZ-induced apoptosis in *miR-519a*-knockdown U87-MG cells (Fig. 3c, dc, d). In particular, 3-MA strongly inhibited the transformation of LC3-I into LC3-II and decreased the TMZ-induced activation of caspase-3 in *miR-519a*-overexpressing U87-MG/TMZ cells (Fig. 3f). Meanwhile, rapamycin significantly promoted the LC3-II accumulation and increased the TMZ-induced activation of caspase-3 in *miR-519a*-knockdown U87-MG cells (Fig. 3f). These findings strongly suggested that the enhanced apoptosis of GBM cells induced by the combination of *miR-519a* and TMZ is dependent on autophagy.

### miR-519a induced autophagy through modification of STAT3 expression

**Fig. 4:**
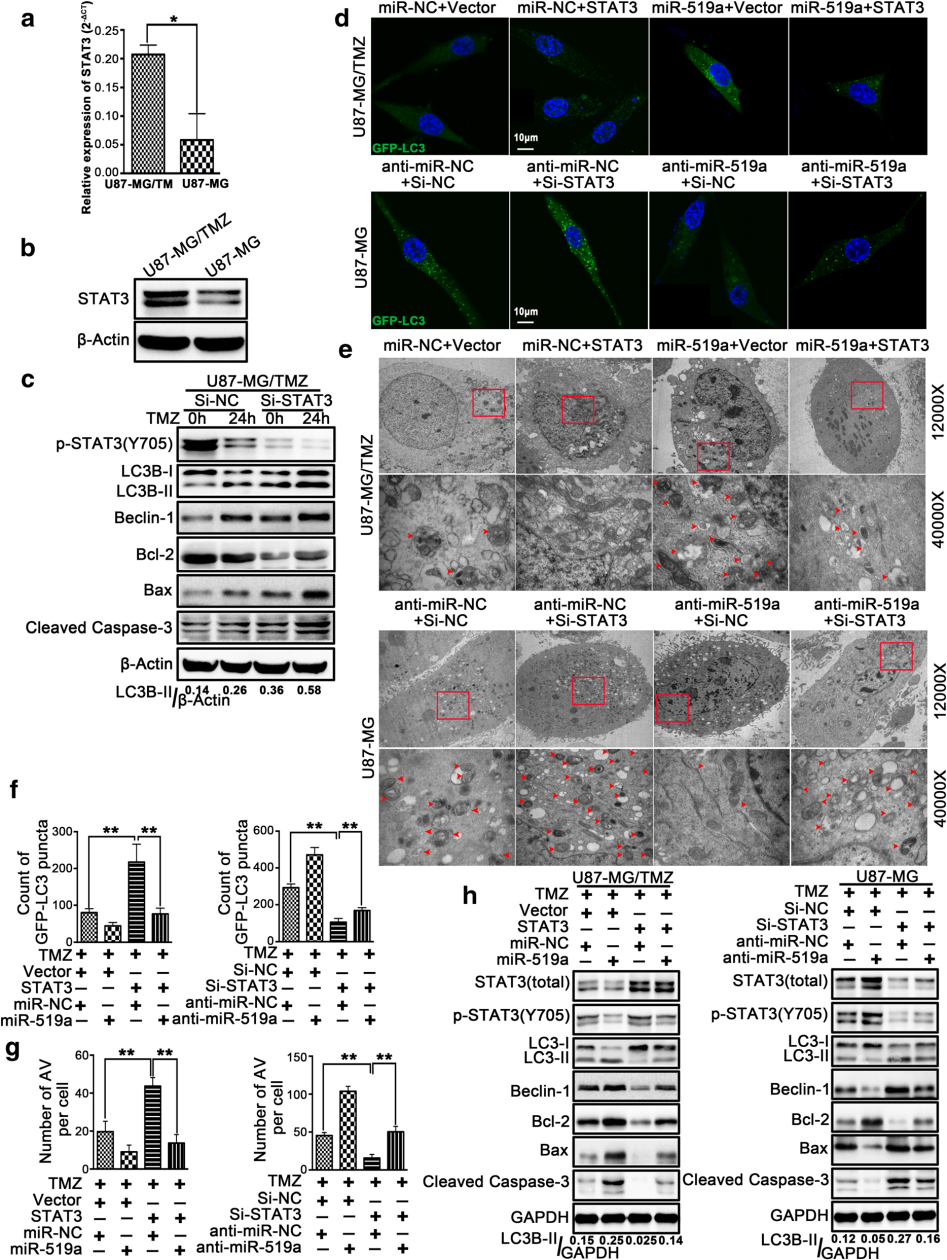
*miR-519a* induced autophagy through the modification of STAT3 expression. The expression levels of STAT3 in U87-MG/TMZ and U87-MG cells were evaluated by qRT-PCR (**a**) and Western blotting (**b**). U87-MG/TMZ cells were transfected with STAT3 siRNA or negative control siRNA and were subjected to immunoblotting with the indicated antibodies (**c**). STAT3 knockdown affected GFP-LC3 dot aggregation (**d**, **f**). Knockdown of STAT3 affected the number of autophagic vacuoles (**e**, **g**). AV (autophagic vacuoles) = autophagosomes and lysosomes. Effects of STAT3 on *miR-519a*-enhanced autophagy and apoptosis were analyzed by immunoblotting in the indicated cells (**h**). *p < 0.05, **p < 0.01

We have previously demonstrated that *miR-519a* can target STAT3 in GBM [21] and speculated that STAT3 may be involved in *miR-519a*-enhanced autophagy after TMZ treatment. Thus, qRT-PCR and Western blotting analysis were performed to determine the expression levels of STAT3 in both U87-MG and U87-MG/TMZ cells. The results indicated that STAT3 expression was increased in U87-MG/TMZ cells (Fig. 4a, b).

A total of three siRNAs were constructed, and the one with the most efficient knockdown of STAT3 was chosen (Additional file 9: Figure S7). Transfection with STAT3 siRNA inhibited both basal autophagy and TMZ-induced autophagy, thus suggesting that STAT3 may be involved during the induction of autophagy in GBM cells (Fig. 4c). Furthermore, we constructed STAT3-expressing plasmids and siRNA to evaluate whether co-transfection with *miR-519a* mimic or inhibitor can counteract the effect of STAT3-expressing plasmids or STAT3 siRNA in GBM cells.

Ectopic expression of STAT3 significantly attenuated the effects of *miR-519a* on autophagy induction in U87-MG/TMZ cells, while suppression of STAT3 can stimulate anti-*miR-519a*-dependent autophagy in U87-MG cells. Moreover, ectopic expression of STAT3 may decrease the number of GFP-LC3 dots (Fig. 4d, f) and autophagic vacuoles (Fig. 4e, g) induced by *miR-519a*. The results of Western blotting analysis also revealed that STAT3 significantly attenuated the *miR-519a*-enhanced autophagy and apoptosis in GBM cells (Fig. 4h). Collectively, these findings suggested that STAT3 was critical for *miR-519a*-enhanced autophagy after TMZ treatment in GBM cells.

### miR-519a sensitized GBM cells to TMZ treatment in vivo

**Fig. 5:**
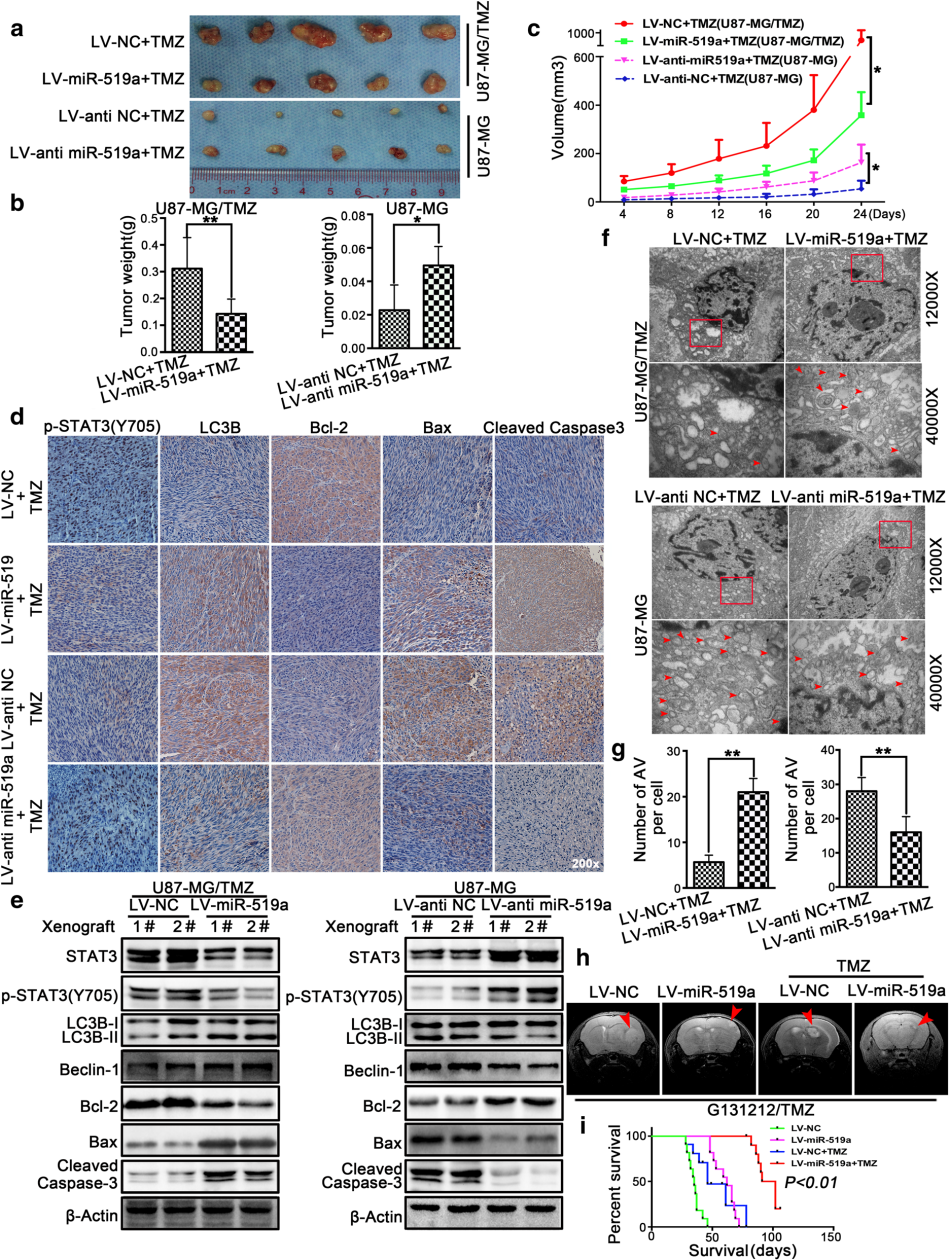
*miR-519a* enhanced the antitumor efficacy of TMZ in vivo. The dissected tumors were collected at the end of drug administration (**a**), and tumor weight was measured (**b**). The tumor volume was calculated as 4/3 × 3.14 × radius (mm)^3^ (**c**). Immunohistochemical analysis (**d**) and Western blot analysis (**e**) of phospho-STAT3, LC3B, Bcl-2, Bax, and cleaved caspase-3 levels in xenograft tumors. Xenograft tumors were subjected to TEM. AV (autophagic vacuoles) = autophagosomes and lysosomes (**f**, **g**). Coronal T2-weighted MRI of tumors acquired from patient-derived GBM cells (G131212/TMZ) from one animal in each treatment group (red arrow) in the brain samples on day 24 after treatment (**h**). The survival of mice with orthotopic tumors was measured by Kaplan-Meier survival curves (**i**). *p < 0.05, **p < 0.01

To investigate the effects of *miR-519a* in vivo, we established U87-MG/TMZ cells with stable overexpression of *miR-519a* and U87-MG cells with stable knockdown of *miR-519a*. Both U87-MG/TMZ and U87-MG cells were infected with LV-miR-519a and LV-anti-miR-519a before they were applied to a subcutaneous xenograft model. After treatment with TMZ, tumors derived from *miR-519a*-overexpressing U87-MG/TMZ cells grew more slowly and had lower tumor weight than those derived from cells harboring empty vector. Meanwhile, downregulation of *miR-519a* expression by LV-anti-miR-519a suppressed the chemosensitivity of U87-MG cells to TMZ (Fig. 5a–c).

Immunohistochemical analysis revealed that *miR-519a* increased the levels of LC3B, Bax, and cleaved caspase-3 and decreased the levels of phospho-STAT3 and Bcl-2, or vice versa in xenograft tumors (Fig. 5d). TEM results demonstrated that forced expression of *miR-519a* increased the numbers of autophagic vacuoles in GBM tissues, whereas knockdown of *miR-519a* decreased the numbers of autophagic vacuoles after TMZ treatment (Fig. 5f, g). These in vivo findings suggested that the chemosensitizing effect of *miR-519a* may be contributed to autophagy induction.

Additionally, the antitumor efficacy of *miR-519a* was examined in an orthotopic G131212/TMZ xenograft model. MRI results on day 24 revealed lower tumor volumes in *miR-519a*-overexpressing tumor cells than in control (Fig. 5h). Further results from Kaplan-Meier survival analysis showed that *miR-519a* can improve the survival time after tumor cell implantation (Fig. 5i). These in vivo results supported that *miR-519a* can sensitize GBM cells to TMZ.

### miR-519a was associated with chemoresistance of GBM

**Fig. 6:**
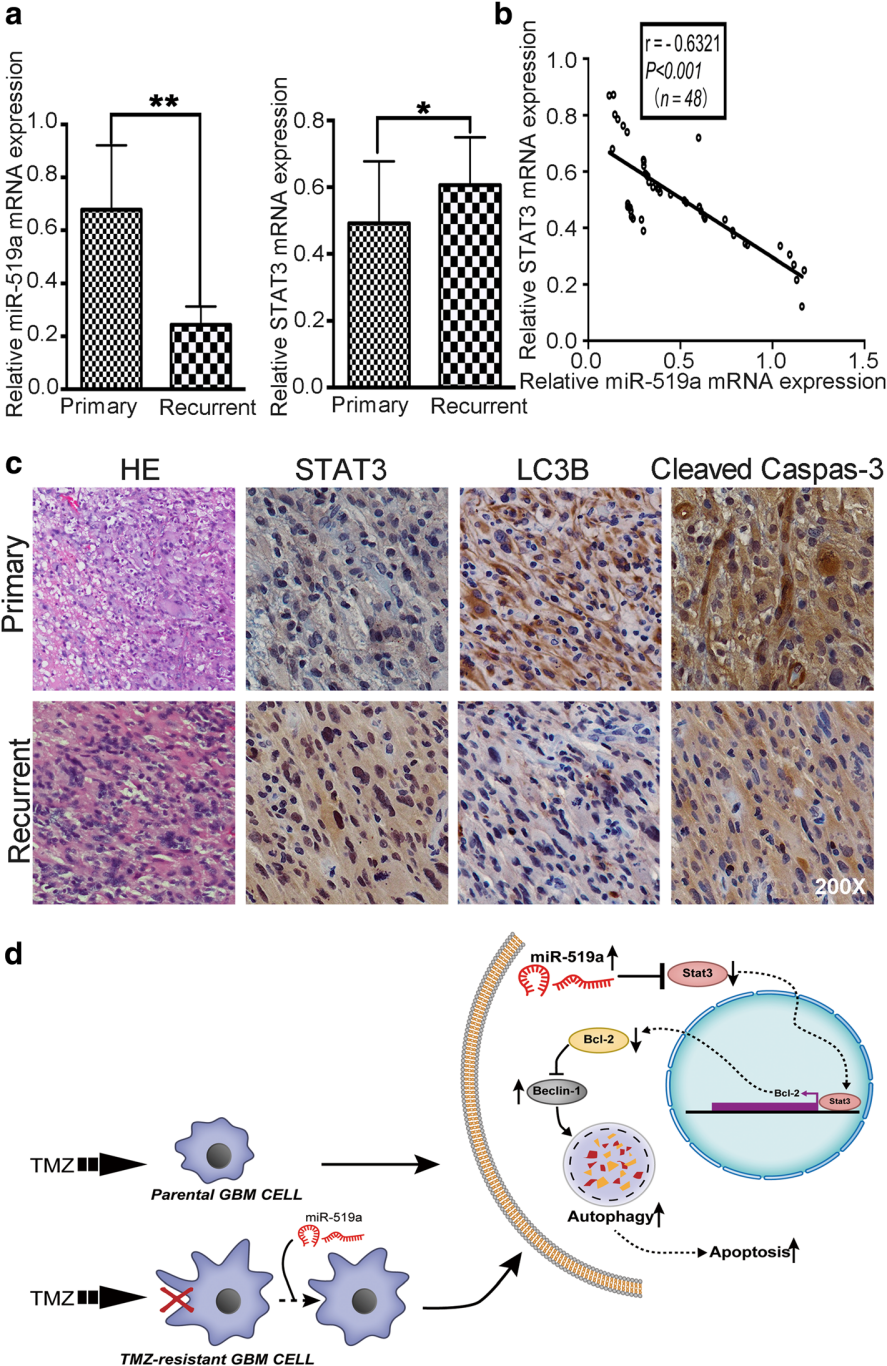
*miR-519a* was associated with chemoresistance. **a** *miR-519a* expression was assessed in primary (*n* = 24) and recurrent GBM tissue samples (*n* = 24). **b** Expression levels of *miR-519a* were inversely correlated with *STAT3* mRNA in tissue samples, as measured by linear regression analysis. **c** Expression of STAT3, LC3B, and cleaved caspase-3 in primary and recurrent GBM tissue samples were determined by immunochemical staining (magnification × 200). **d** Schematic illustration of the mechanisms underlying *miR-519a* induced chemosensitivity to TMZ. Pointed arrows and blunted arrows indicate both activation and repression, respectively. *p < 0.05, **p < 0.01

To further evaluate the clinical role of *miR-519a* in clinical samples (Additional file 10: Table S3), we performed qRT-PCR assays to detect the expression levels of *miR-519a* and STAT3 in brain tissues from patients with primary and recurrent GBM. We identified downregulation of *miR-519a* and upregulation of STAT3 in recurrent GBM tissues compared to primary GBM tissues (Fig. 6a). A significant inverse correlation was found between *miR-519a* and STAT3 expression levels (Fig. 6b). Similarly, the results from immunohistochemical staining showed the increased STAT3 expression and reduced LC3B and cleaved caspase-3 levels in recurrent GBM tissues compared to primary GBM tissues (Fig. 6c). Therefore, these differences strongly suggested an apparent association between *miR-519a*, STAT3, and LC3B in GBM patients.

## Discussion

GBM is the most common type of malignant brain tumor [30]. Even after multimodality treatment including radical surgery, radiation, and chemotherapy, the median survival time of GBM is approximately 1 year from diagnosis. De novo and acquired resistance to TMZ in GBM cells have emerged as a challenging problem in clinical practice [31]. Therefore, identifying the mechanisms underlying TMZ chemoresistance shed light on a novel combination therapy strategy to circumvent acquired resistance in GBM patients. Numerous studies have reported that miRNA dysfunction may be involved in tumor progression and therapeutic resistance [20, 32, 33]. Moreover, the therapeutic potential of miRNAs in cancer, either alone or in combination with conventional drugs, has been demonstrated in several published studies (reviewed in [34]). Previously, we reported that *miR-519a* is downregulated in GBM cells, and overexpression of *miR-519a* may suppress GBM cell proliferation [21]. However, the pivotal role of *miR-519a* in the modulation of TMZ sensitivity is still not fully understood. In this study, we demonstrated that the expression of *miR-519a* was reduced in chemoresistant GBM tissues and TMZ-resistant cells, thus suggesting that low levels of *miR-519a* were associated with TMZ resistance. In addition, our results showed that *miR-519a* enhanced chemosensitivity in GBM cells, mainly through TMZ-induced autophagy and apoptosis.

Both prosurvival and prodeath roles of autophagy have been proposed in GBM cells in response to metabolic and therapeutic stress and are dependent on the cellular context and duration or degree of stress stimuli. Moreover, accumulating evidence has revealed a correlative relationship between chemoresistance and reduced autophagic activity in GBM cells [35–37]. Consistent with previous studies, we found that the basal level of autophagy was lower in TMZ-resistant U87-MG/TMZ cells than in parental GBM cells, suggesting an inverse correlation between reduced autophagy and chemoresistance (Additional file 11). Therefore, restoration of autophagic activity in resistant GBM cells may be a promising strategy to overcome chemoresistance and improve the effectiveness of chemotherapy. In this study, we demonstrated that the enhanced autophagy by forced *miR-519a* expression can sensitize GBM cells to TMZ. Additionally, these effects were attenuated by co-treatment with autophagic blockers (3-MA), suggesting the involvement of autophagic pathway. These results are consistent with previous research demonstrating that autophagy induction can lead to the suppression of GBM cell growth [10–14]. Furthermore, the combination of *miR-519a* and TMZ induced prodeath autophagy, suggesting that the autophagic response of GBM cells to TMZ can be modified when administered in combination with other antitumor agents.

Because the autophagy-related signal pathway is complex, additional studies are needed to elucidate the mechanisms of cell autophagy regulation. Beclin-1 may bind to and be inhibited by Bcl-2 protein to prevent cell autophagy [38, 39]. Furthermore, STAT3/Bcl-2/Beclin-1 signaling is associated with the induction of autophagy. Previous reports have showed that STAT3 has the ability to transcriptionally activate the apoptosis-inhibitory protein BCL2, which also inhibits the induction of autophagy by dissociating the Bcl-2/Beclin-1 complex. Upon activation, STAT3 upregulates BCL2 expression and consequently leads to autophagy inhibition [33, 40, 41]. In our study, transfection with STAT3 siRNA also inhibited both basal autophagy and TMZ-induced autophagy, suggesting that STAT3 was involved in the induction of autophagy in GBM. Moreover, dysfunction of miRNAs can modulate autophagy through a variety of mechanisms in GBM [20, 32, 33]. We previously demonstrated that *miR-519a* functions as a tumor suppressor in glioma by targeting STAT3 [21]; therefore, we hypothesized that *miR-519a*-mediated prodeath autophagy may occur via targeting of STAT3 to sensitize U87-MG/TMZ cells to TMZ. Indeed, in this study, we found that *miR-519a* promoted the autophagy of GBM cells by enhancing dissociation of the Bcl-2/Beclin-1 complex and enhanced therapeutic efficacy in vivo and in vitro. We further improved our understanding of the molecular basis of *miR-519a* in GBM. Advances in molecular biology have promoted our understanding of the molecular basis of GBM and provide tools with which to improve therapy. There are many tools, including decoy oligonucleotides/antisense oligonucleotide/RNA interference and guanine-rich oligonucleotides, that have been very promising in modulating STAT3 pathway and facilitating the development of new drugs for clinical applications [42]. miR-519a, as a small molecule, may be a favorable candidate for analysis in clinical trials.

More recently, several studies have suggested that autophagy plays a prodeath role in GBM cells treated with chemotherapeutic agents, by enhancing autophagy-mediated apoptosis instead of autophagic cell death [41–43]. Mu et al. [43] observed an induced autophagy and apoptosis in GBM cells treated with a combination treatment of β-elemene and gefitinib. Bak et al. [44] reported that enhanced autophagy contributes to the synergistic effects of vitamin D in TMZ-based GBM chemotherapy. Peng-Hsu et al. [45] found that *miR-128* promotes apoptotic death in glioma cells through non-protective autophagy formation. Our results, in agreement with previous findings [41–43], showed that autophagy inhibition by 3-MA may reduce apoptosis during combined treatment of *miR-519a* and TMZ, whereas rapamycin-induced autophagy can enhance apoptosis following combined treatment of anti-*miR-519a* and TMZ. Nevertheless, emerging evidence has suggested a crosstalk between autophagic and apoptotic pathways [46].

Since the autophagy-related pathway network can be complex, additional studies are required to elucidate the molecular mechanisms underlying cell autophagy. STAT3/Bcl-2/Beclin-1 signaling has been proposed to be associated with autophagy induction. Notably, Beclin-1 may be bound to or inhibited by Bcl-2 protein in order to prevent cell autophagy [38, 39]. STAT3 is able to induce BCL2 transcriptional activation, which inhibits the induction of autophagy by dissociating the Bcl-2/Beclin-1 complex [33, 40, 41]. In this study, transfection with STAT3 siRNA inhibited both basal autophagy and TMZ-induced autophagy, suggesting that STAT3 signaling pathway is involved in TMZ-induced autophagy in GBM cells. Additionally, dysfunction of miRNAs can modulate autophagy in GBM cells through various mechanisms [20, 32, 33]. We previously demonstrated that *miR-519a* functions as a tumor suppressor in glioma by targeting STAT3 [21]. In the present study, we confirmed that *miR-519a* sensitized U87-MG/TMZ cells to TMZ and triggered autophagy-mediated apoptosis via STAT3 pathway. Indeed, we found that *miR-519a* promoted the autophagy of GBM cells via dissociation of Bcl-2/Beclin-1 complex. These results significantly improved our understanding of the molecular basis of *miR-519a* in GBM cells with TMZ resistance.

## Conclusions

The results of this study suggested that *miR-519a* may hold a great potential to overcome TMZ chemoresistance in GBM. In vivo and in vitro analysis clearly indicated that *miR-519a* increased TMZ sensitivity by promoting GBM cell apoptosis through autophagy. Additionally, overexpression of *miR-519a* can induce autophagy via the inhibition of STAT3/Bcl-2 signaling pathway. Therefore, *miR-519a* in combination with TMZ therapy could render a more effective therapeutic approach for GBM.

## Additional files

Additional file 1: **Table S1.** Sequences of siRNAs and miRNA used in this study. (DOCX 58 kb)

Additional file 2: **Table S2.** List of primer sequences used in this study. (DOCX 48 kb)

Additional file 3: **Figure S1.** Determination of cell growth rates by using doubling time assay. U87-MG and U87-MG/TMZ cells displayed doubling times of 37.1 and 29.2 h, respectively. Each bar represents the mean ± s.d. of three independent experiments. (TIF 107 kb)

Additional file 4: **Figure S2.** TMZ enhanced the expression of *miR-519a* in U87-MG cells but showed no effect on U87-MG/TMZ cells. U87-MG cells and U87-MG/TMZ cells were treated with different concentrations of TMZ for 24 h or with 200 μM TMZ for the indicated times. The expression of *miR-519a* was measured by qRT-PCR. a TMZ enhanced the levels of *miR-519a* in U87-MG cells in a concentration-dependent manner. b TMZ induced *miR-519a* upregulation in a time-dependent manner. Each bar represents the mean ± s.d. of three independent experiments. *p < 0.05, **p < 0.01, NS > 0.05 vs. control group. (TIF 1152 kb)

Additional file 5: **Figure S3.** *miR-519a* sensitized GBM cells to TMZ treatment. a Cell viability of U87-MG/TMZ and U87-MG cells transfected with pCMV-miR-519a or *miR-519a* sponge and then treated with or without TMZ at various concentrations (or times). b Colony formation in U87-MG/TMZ and U87-MG cells transfected with pCMV-miR-519a or *miR-519a* sponge and then treated with or without TMZ at various concentrations (or times). Each bar represents the mean ± s.d. of three independent experiments. NS > 0.05, *p < 0.05, **p < 0.01. (TIF 4190 kb)

Additional file 6: **Figure S4.** *miR-519a* enhanced radiosensitivity in GBM cells. a Cell viability of GBM cells after treatment. Each bar represents the mean ± standard deviation of three independent experiments. b Clonogenic survival of GBM cells transfected with *miR-519a* or anti-*miR-519a*. Each bar represents the mean ± s.d. of three independent experiments. *p < 0.05, **p < 0.01. (TIF 1670 kb)

Additional file 7: **Figure S5.** Cellular viability assay for TMZ-sensitive and -resistant cells. a U87-MG/TMZ and U87-MG cells were cultured in normal medium, with low glucose (LG), or in the presence of chloroquine (CQ). b The cell viability of U87-MG/TMZ and U87-MG treated with different concentrations of rapamycin for 72 h. The cell viability for a and b was evaluated by MTT assays. Data represent the mean (± standard deviation) of three independent experiments. *p < 0.05, **p < 0.01, NS > 0.05 vs. control group. (TIF 816 kb)

Additional file 8: **Figure S6.** Effects of 3-MA, rapamycin(Rapa), and/or their combination with TMZ on the viability of U87-MG/TMZ and U87-MG cells. a Rapamycin is not able to affect the viability of U87MG/TMZ cells. b 3-MA is not able to affect the viability of U87MG. Each bar represents the mean ± s.d. of three independent experiments. NS > 0.05,*p < 0.05. (TIF 538 kb)

Additional file 9: **Figure S7.** The knockdown efficiency of siSTAT3. Cells transfected with STAT3 siRNAs (NS, #1, #2, or #3) were treated with or without TMZ (400 μm) for 48 h. qRT-PCR (a) and Western blot analysis (b) for the respective target genes were carried out 48 h after transfection. Immunoblots (c) of the extracts for the indicated proteins in U87-MG cells. GAPDH was used as a loading control for Western blots. **p < 0.01 vs. Si-NC group. (TIF 599 kb)

Additional file 10: **Table S3.** Clinical information of patients with recurrent GBM. (DOCX 105 kb)

Additional file 11: Supplemental Material and Methods. (DOCX 104 kb)

## Abbreviations

3′-UTR: 3′-Untranslated region
AV: Autophagic vacuole
GBM: Glioblastoma
GFP: Green fluorescent protein
miR-519a: MicroRNA-519a
miRNA: MicroRNA
PCR: Polymerase chain reaction
TEM: Transmission electron microscopy
TMZ: Temozolomide
