# Supplementary material for: miR-519a enhances chemosensitivity and promotes autophagy in glioblastoma by targeting STAT3/Bcl2 signaling pathway
Source: J Hematol Oncol. 2018 May 29;11:70. doi: 10.1186/s13045-018-0618-0 (PMC5975545; doi:10.1186/s13045-018-0618-0)
Supplement: Supplementary file 1 — Table S1. Sequences of siRNAs and miRNA used in this study. (DOCX 58 kb) [file 13045_2018_618_MOESM1_ESM.docx]

|  | Gene name | Sequence(5′-3′) |
| --- | --- | --- |
| siRNA |  |  |
|  | STAT3 siRNA #1 | TGCATAGGACGGAATGAAC |
|  | STAT3 siRNA #2 | GCGACGUGAGGUAUAUGAC |
|  | STAT3 siRNA #3 | GGAACAACAUUAGAACAGC |
|  | Negative control siRNA | UUCUCCGAACGUGUCACGUTT |
| miRNA |  |  |
|  | *miR-519a* mimic | AAAGUGCAUCCUUUUAGAGUGU |
|  | mimic-NC | UUCUCCGAACGUGUCACGUTT |
|  | *miR-519a* inhibitor | AAAGTGCATCCTTTTAGAGTGT |
|  | inhibitor-NC | CAGUACUUUUGUGUAGUACCA |
