# Supplementary material for: miR-519a enhances chemosensitivity and promotes autophagy in glioblastoma by targeting STAT3/Bcl2 signaling pathway
Source: J Hematol Oncol. 2018 May 29;11:70. doi: 10.1186/s13045-018-0618-0 (PMC5975545; doi:10.1186/s13045-018-0618-0)
Supplement: Supplementary file 2 — Table S2. List of primer sequences used in this study. (DOCX 48 kb) [file 13045_2018_618_MOESM2_ESM.docx]

|  | Gene name | Sequence(5′-3′) |
| --- | --- | --- |
| qPCR primers |  |  |
|  | miR-519a-F | AAAGTGCATCCTTTTAGAGTGT |
|  | miR-519a-R | GTGCAGGGTCCGAGGTATT |
|  | U6-F | GCGCGTCGTGAAGCGTTC |
|  | U6-R | GTGCAGGGTCCGAGGT |
|  | STAT3-F | ATCACGCCTTCTACA GACTGC |
|  | STAT3-R | CATCCTGGAGATTCTCTACCACT |
|  | GAPDH-F | CCACTCCTCCACCTTTGAC |
|  | GAPDH-R | ACCCTGTTGCTGTAGCCA |

F = Forward primer; R = Reverse primer.
