# Supplementary figures and images for: miR-519a enhances chemosensitivity and promotes autophagy in glioblastoma by targeting STAT3/Bcl2 signaling pathway
Source: J Hematol Oncol. 2018 May 29;11:70. doi: 10.1186/s13045-018-0618-0 (PMC5975545; doi:10.1186/s13045-018-0618-0)

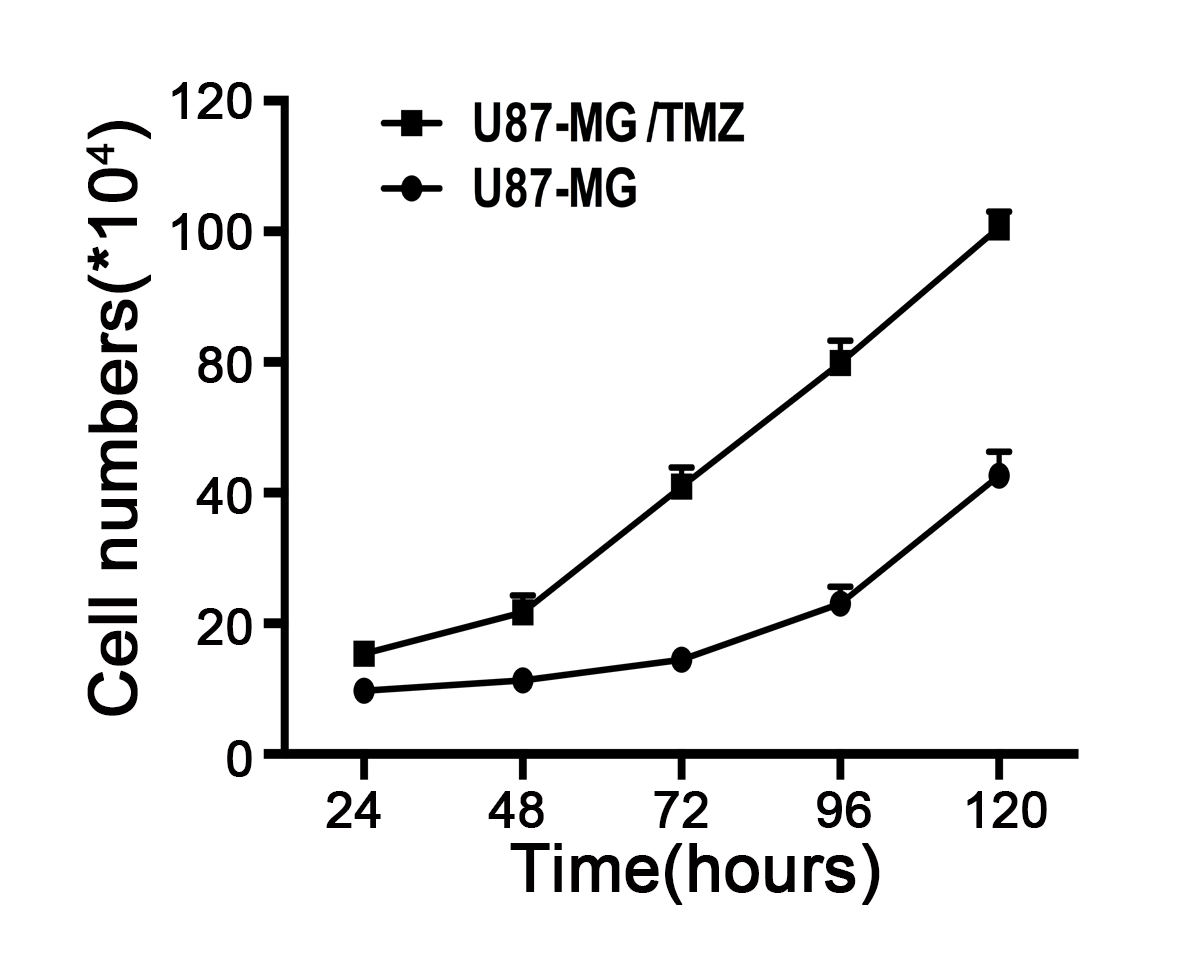

Supplement: Supplementary file 3 — Figure S1. Determination of cell growth rates by using doubling time assay. U87-MG and U87-MG/TMZ cells displayed doubling times of 37.1 and 29.2 h, respectively. Each bar represents the mean ± s.d. of three independent experiments. (TIF 107 kb) [file 13045_2018_618_MOESM3_ESM.tif]

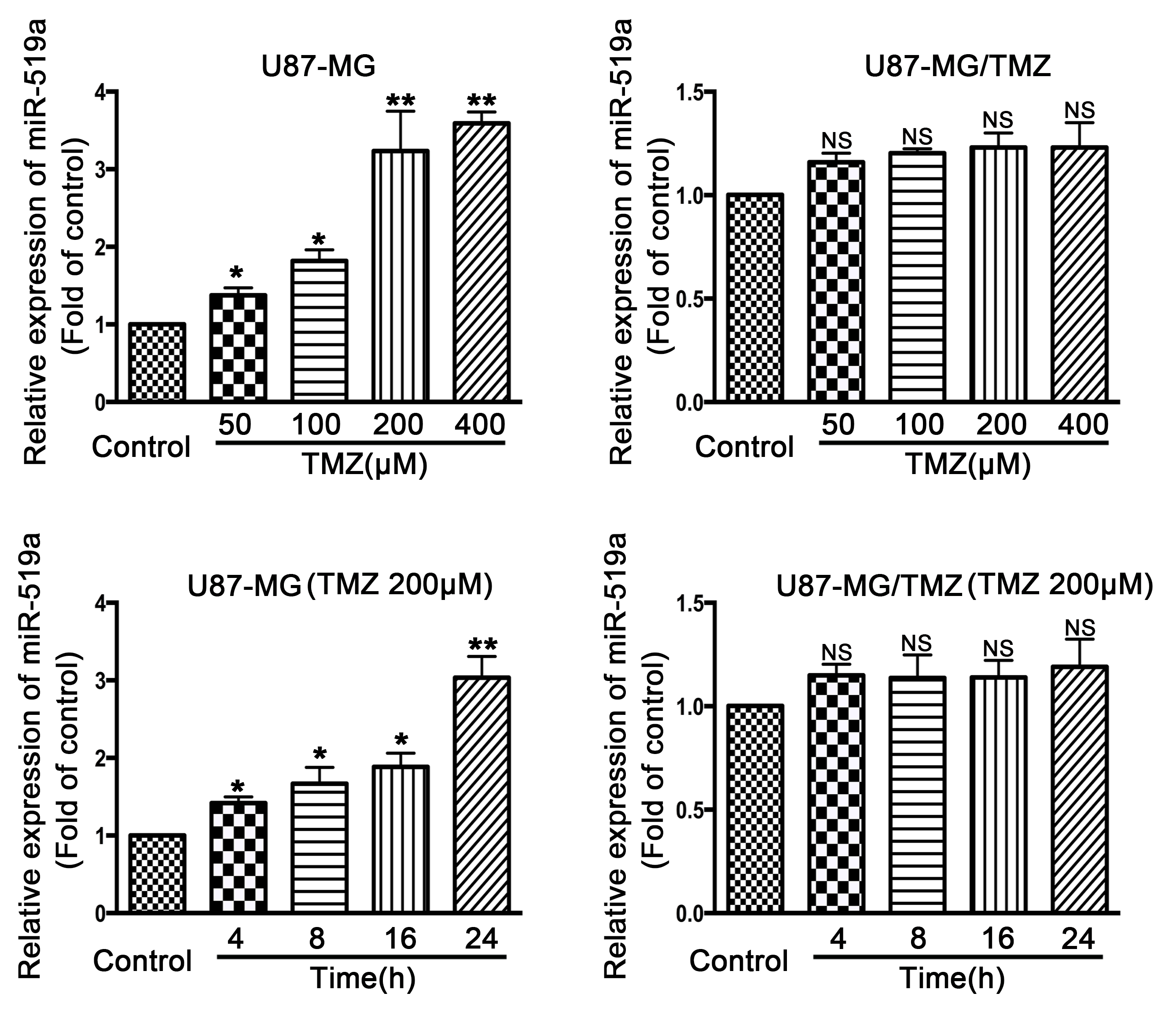

Supplement: Supplementary file 4 — Figure S2. TMZ enhanced the expression of miR-519a in U87-MG cells but showed no effect on U87-MG/TMZ cells. U87-MG cells and U87-MG/TMZ cells were treated with different concentrations of TMZ for 24 h or with 200 μM TMZ for the indicated times. The expression of miR-519a was measured by qRT-PCR. a TMZ enhanced the levels of miR-519a in U87-MG cells in a concentration-dependent manner. b TMZ induced miR-519a upregulation in a time-dependent manner. Each bar represents the mean ± s.d. of three independent experiments. *p < 0.05, **p < 0.01, NS > 0.05 vs. control group. (TIF 1152 kb) [file 13045_2018_618_MOESM4_ESM.tif]

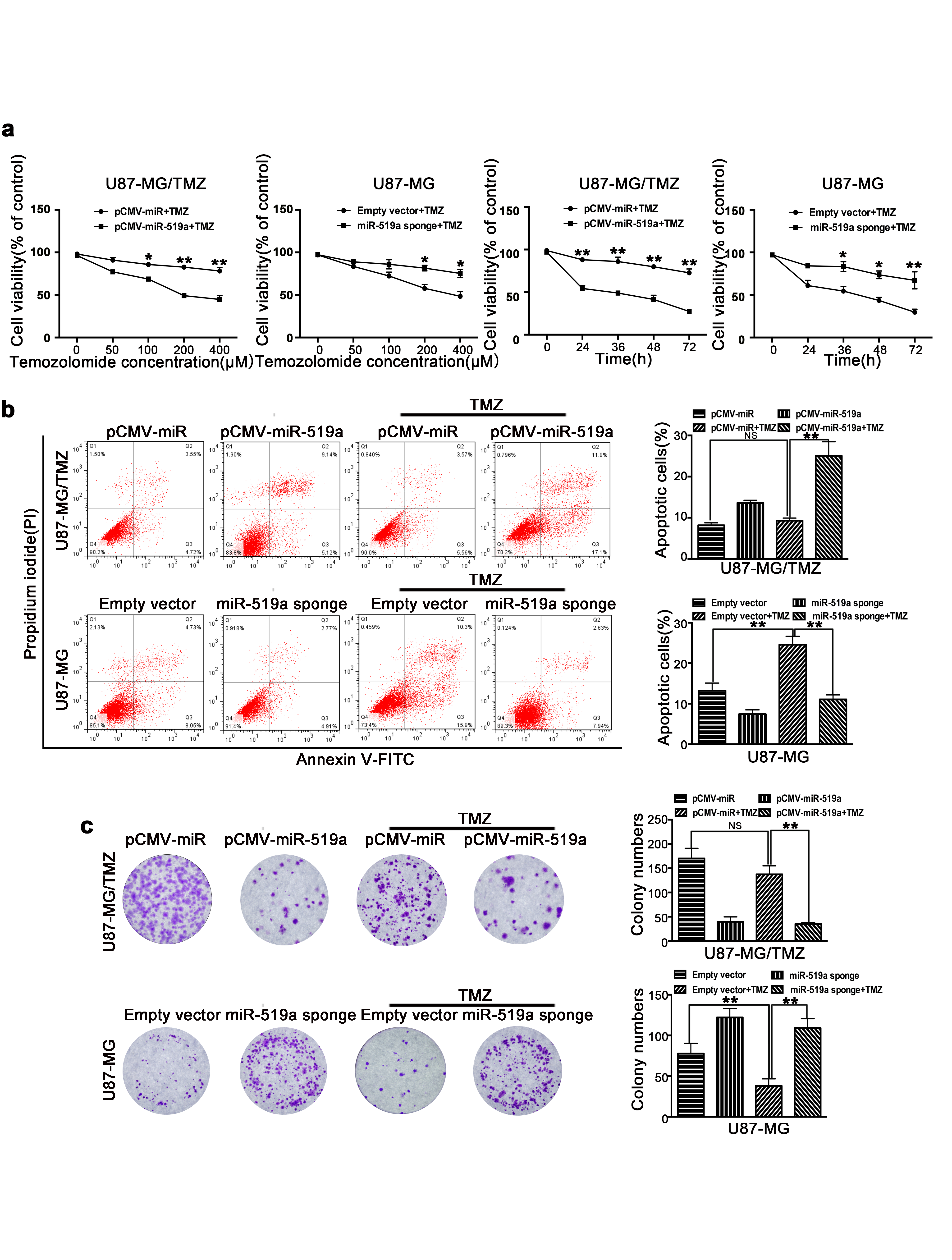

Supplement: Supplementary file 5 — Figure S3. miR-519a sensitized GBM cells to TMZ treatment. a Cell viability of U87-MG/TMZ and U87-MG cells transfected with pCMV-miR-519a or miR-519a sponge and then treated with or without TMZ at various concentrations (or times). b Colony formation in U87-MG/TMZ and U87-MG cells transfected with pCMV-miR-519a or miR-519a sponge and then treated with or without TMZ at various concentrations (or times). Each bar represents the mean ± s.d. of three independent experiments. NS > 0.05, *p < 0.05, **p < 0.01. (TIF 4190 kb) [file 13045_2018_618_MOESM5_ESM.tif]

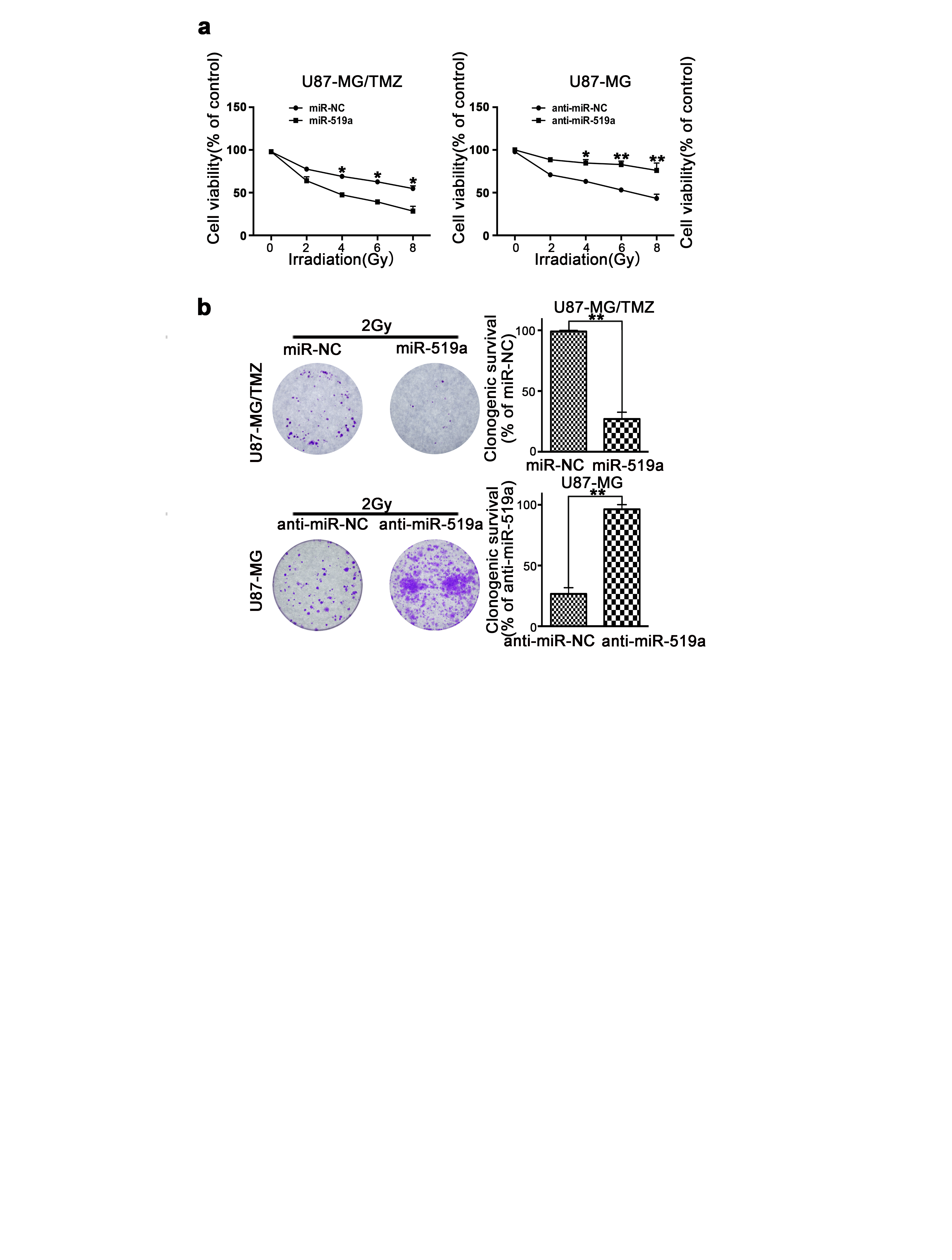

Supplement: Supplementary file 6 — Figure S4. miR-519a enhanced radiosensitivity in GBM cells. a Cell viability of GBM cells after treatment. Each bar represents the mean ± standard deviation of three independent experiments. b Clonogenic survival of GBM cells transfected with miR-519a or anti-miR-519a. Each bar represents the mean ± s.d. of three independent experiments. *p < 0.05, **p < 0.01. (TIF 1670 kb) [file 13045_2018_618_MOESM6_ESM.tif]

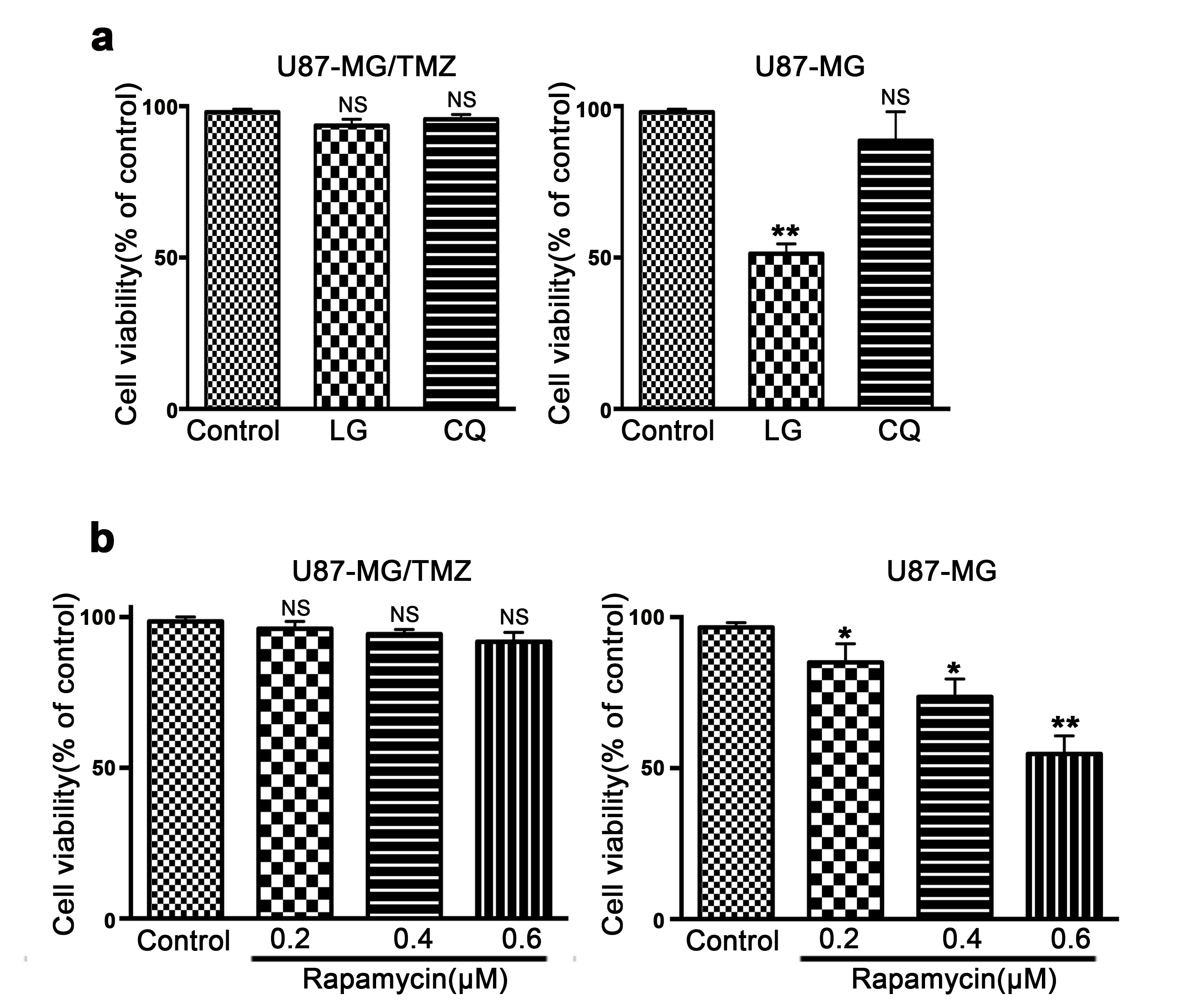

Supplement: Supplementary file 7 — Figure S5. Cellular viability assay for TMZ-sensitive and -resistant cells. a U87-MG/TMZ and U87-MG cells were cultured in normal medium, with low glucose (LG), or in the presence of chloroquine (CQ). b The cell viability of U87-MG/TMZ and U87-MG treated with different concentrations of rapamycin for 72 h. The cell viability for a and b was evaluated by MTT assays. Data represent the mean (± standard deviation) of three independent experiments. *p < 0.05, **p < 0.01, NS > 0.05 vs. control group. (TIF 816 kb) [file 13045_2018_618_MOESM7_ESM.tif]

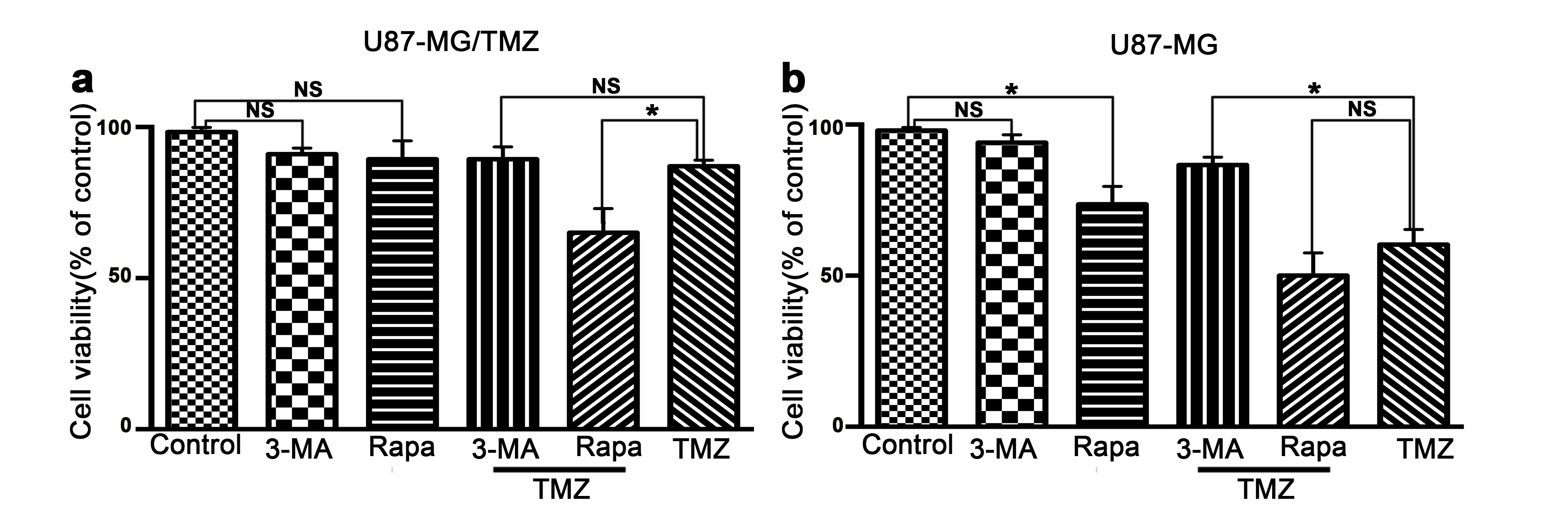

Supplement: Supplementary file 8 — Figure S6. Effects of 3-MA, rapamycin(Rapa), and/or their combination with TMZ on the viability of U87-MG/TMZ and U87-MG cells. a Rapamycin is not able to affect the viability of U87MG/TMZ cells. b 3-MA is not able to affect the viability of U87MG. Each bar represents the mean ± s.d. of three independent experiments. NS > 0.05,*p < 0.05. (TIF 538 kb) [file 13045_2018_618_MOESM8_ESM.tif]

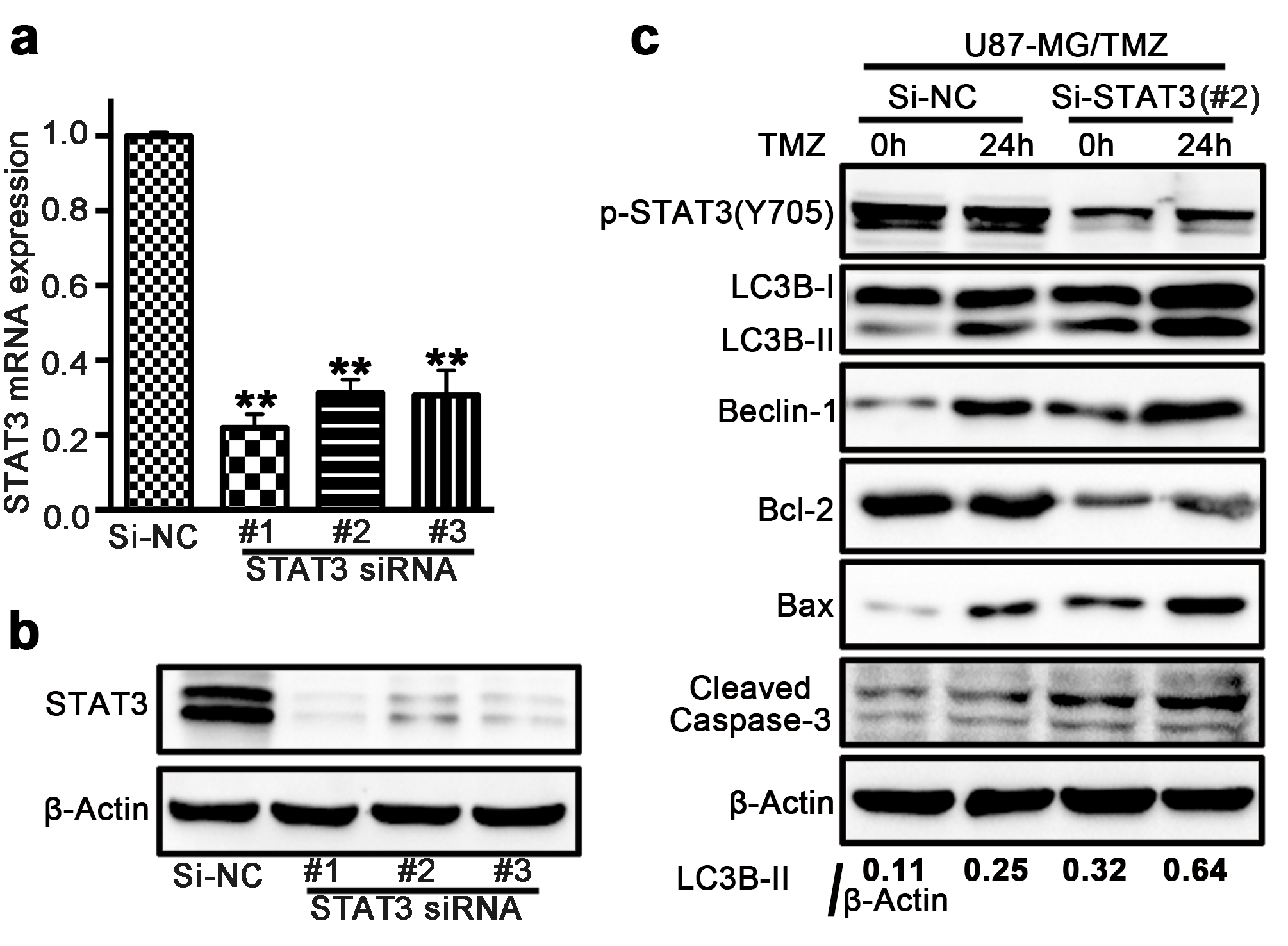

Supplement: Supplementary file 9 — Figure S7. The knockdown efficiency of siSTAT3. Cells transfected with STAT3 siRNAs (NS, #1, #2, or #3) were treated with or without TMZ (400 μm) for 48 h. qRT-PCR (a) and Western blot analysis (b) for the respective target genes were carried out 48 h after transfection. Immunoblots (c) of the extracts for the indicated proteins in U87-MG cells. GAPDH was used as a loading control for Western blots. **p < 0.01 vs. Si-NC group. (TIF 599 kb) [file 13045_2018_618_MOESM9_ESM.tif]
