# Supplementary material for: miR-519a enhances chemosensitivity and promotes autophagy in glioblastoma by targeting STAT3/Bcl2 signaling pathway
Source: J Hematol Oncol. 2018 May 29;11:70. doi: 10.1186/s13045-018-0618-0 (PMC5975545; doi:10.1186/s13045-018-0618-0)
Supplement: Supplementary file 10 — Table S3. Clinical information of patients with recurrent GBM. (DOCX 105 kb) [file 13045_2018_618_MOESM10_ESM.docx]

**Table S1.** Clinicopathological characteristics of tumor samples.

| **Lab annotation** | **Age**  **(Years)** | **Gender** | **Volume**  **(cm3)*** | **Side and Tumor location** | **Therapy Class** | **Tumor resection#** | **Recurrent**  **(Months)** | **Survival**  **(Months)** | **Survival status** | **Pathological type** |
| --- | --- | --- | --- | --- | --- | --- | --- | --- | --- | --- |
| GBM-2 | 68 | Male | 21.8 | Right, Parietal lobe | TMZ Chemoradiation, TMZ Chemo | PTR | 3 | 4 | Dead | IDH-wildtype |
| **GBM-6** | **46** | **Female** | **48.6** | **Right, Frontal lobe** | **TMZ Chemoradiation, TMZ Chemo** | **GTR** | **7** | **16** | **Dead** | **IDH-mutant** |
| GBM-8 | 45 | Male | 38.3 | Right, Temporal lobe | TMZ Chemoradiation, TMZ Chemo | STR | 4 | 4 | Dead | IDH-mutant |
| GBM-10 | 52 | Female | 22.6 | Right, Frontal lobe | TMZ Chemoradiation, TMZ Chemo | STR | 5 | 5 | Dead | IDH-wildtype |
| GBM-12 | 38 | Female | 72.1 | Right, Occipital lobe | TMZ Chemoradiation, TMZ Chemo | GTR | 6 | 6 | Dead | IDH-wildtype |
| GBM-14 | 46 | Male | 14.5 | Right, Thalamus | TMZ Chemoradiation, TMZ Chemo | PTR | 7 | 10 | Dead | IDH-wildtype |
| **GBM-15** | **58** | **Male** | **24.8** | **Left, Temporal lobe** | **TMZ Chemoradiation, TMZ Chemo** | **GTR** | **12** | **30** | **Dead** | **IDH-mutant** |
| GBM-22 | 52 | Male | 10.6 | Left, Frontal lobe | TMZ Chemoradiation, TMZ Chemo | GTR | 9 | 10 | Dead | IDH-wildtype |
| GBM-27 | 42 | Male | 15.5 | Right, Parietal lobe | TMZ Chemoradiation, TMZ Chemo | STR | 15 | 18 | NA | IDH-wildtype |
| GBM-28 | 41 | Male | 94.4 | Left, Frontal lobe | TMZ Chemoradiation, TMZ Chemo | STR | 16 | 22 | NA | IDH-wildtype |
| **GBM-31** | **48** | **Male** | **9** | **Right, Temporal lobe** | **TMZ Chemoradiation, TMZ Chemo** | **GTR** | **20** | **33** | **Dead** | **IDH-mutant** |
| GBM-32 | 48 | Female | 9.1 | Right, Brain stem | TMZ Chemoradiation, TMZ Chemo | PTR | 22 | 22 | Dead | IDH-wildtype |
| GBM-36 | 56 | Male | 60.4 | Left, Temporal lobe | TMZ Chemoradiation, TMZ Chemo | STR | 8 | 13 | Dead | **IDH-mutant** |
| **GBM-38** | **62** | **Female** | **24** | **Left, Occipital lobe** | **TMZ Chemoradiation, TMZ Chemo** | **GTR** | **18** | **19** | **Dead** | **IDH-mutant** |
| GBM-41 | 60 | Female | 25.5 | Right, Temporal lobe | TMZ Chemoradiation, TMZ Chemo | GTR | 9 | 9 | NA | IDH-wildtype |
| **GBM-43** | **61** | **Male** | **39.2** | **Left, Thalamus** | **TMZ Chemoradiation, TMZ Chemo** | **GTR** | **20** | **28** | **Dead** | **IDH-mutant** |
| GBM-49 | 57 | Male | 60.4 | Left, Temporal lobe | TMZ Chemoradiation, TMZ Chemo | STR | 15 | 17 | Dead | IDH-wildtype |
| **GBM-58** | **28** | **Female** | **21.7** | **Right, Temporal lobe** | **TMZ Chemoradiation, TMZ Chemo** | **GTR** | **19** | **20** | **Dead** | **IDH-wildtype** |
| **GBM-60** | **32** | **Male** | **56.1** | **Right, Thalamus** | **TMZ Chemoradiation, TMZ Chemo** | **PTR** | **22** | **28** | **Dead** | **IDH-wildtype** |
| GBM-61 | 49 | Male | 3.5 | Median, Brain stem | TMZ Chemoradiation, TMZ Chemo | PTR | 15 | 19 | Dead | IDH-wildtype |
| **GBM-62** | **52** | **Female** | **11.2** | **Left, Thalamus** | **TMZ Chemoradiation, TMZ Chemo** | **STR** | **17** | **17** | **Dead** | **IDH-wildtype** |
| GBM-64 | 50 | Female | 21.5 | Left, Temporal lobe | TMZ Chemoradiation, TMZ Chemo | STR | 16 | 18 | Dead | IDH-wildtype |
| GBM-76 | 48 | Female | 19.6 | Right, Temporal lobe | TMZ Chemoradiation, TMZ Chemo | GTR | 10 | 10 | Dead | IDH-wildtype |
| GBM-83 | 46 | Male | 19.3 | Right, Thalamus | TMZ Chemoradiation, TMZ Chemo | STR | 13 | 20 | Dead | IDH-wildtype |

*, Tumor volume was measured according to the T1WI scan before surgery using the Coniglobus formula as **“a × b × c / 2”**, which is widely used to evaluate the volume of intracranial hemorrhage. **“a”** indicates the maximal diameter in the tumor cross section,

**“b”** indicates the maximal diameter perpendicular to “a”, and **“c”** indicates the slice thickness of the tumor.

#, The degree of tumor resection. **GTR**, gross total tumor removal, indicating no residual tumor in the postsurgical MRI; **STR**, subtotal tumor removal, indicating that more than 90% of tumor was removed;

**PTR**, partial tumor removal, indicating that less than 90% of the tumor was removed.
