# Supplementary material for: miR-519a enhances chemosensitivity and promotes autophagy in glioblastoma by targeting STAT3/Bcl2 signaling pathway
Source: J Hematol Oncol. 2018 May 29;11:70. doi: 10.1186/s13045-018-0618-0 (PMC5975545; doi:10.1186/s13045-018-0618-0)
Supplement: Supplementary file 11 — Supplemental Material and Methods. (DOCX 104 kb) [file 13045_2018_618_MOESM11_ESM.docx]

**Supplemental Material and Methods**

*Irradiation assays*

Radiosensitivity of the miR-519a overexpressing cells was assessed using Colony-formation assay and MTT cell viability assay.At 48 hr after transfection with oligonucleotides, increasing numbers of cells were seeded on six-well plates in triplicate and exposed to a range of radiation doses (0-8Gy). Irradiation was performed with an X-ray generator (120 kV, 22.7 mA, variable time; GE Inspection Technologies, Hürth, Germany). Twelve days after irradiation, surviving colonies were stained with 0.1% crystal violet and counted. The clogenic survival was calculated as: (number of colonies/number of cells plated)_control_/(number of colonies/number of cells plated)_treated_. The treated cells were seeded in a 96-well plate and incubated for five days. The MTT assay was then carried out as above and measurements expressed relative to the control cells.

*Doubling time of U87-MG/TMZ and U87-MG*

The cells were seeded at a density of 3x104 cells on 6-well culture dishes. After serum starvation for 24 h, the cells were counted every 24 h. The cells were trypsinized and the number of viable cells was counted. The doubling-time was calculated from the cell growth curve over 5 days using the following equation: Doubling time = (final time - initial time) x [log 2/log(final cell number) - log(initial cell number)].
